# Primary Atypical Meningioma of the Nasal Cavity: A Case Report and Review of the Literature

**DOI:** 10.1155/2018/7541892

**Published:** 2018-02-26

**Authors:** Leison Maharjan, Yogesh Neupane, Bibhu Pradhan

**Affiliations:** Department of ENT-HNS, Institute of Medicine (IOM), Kathmandu, Nepal

## Abstract

**Background:**

Meningioma is a central nervous system tumor that typically arises in proximity to meninges. Extracranial primary atypical meningioma of sinonasal tract is a rare one.

**Methods:**

We discuss the clinical, radiological, and histological presentation of an elderly female with primary atypical meningioma of the nasal cavity, which was excised via endoscopic endonasal approach.

**Results:**

There was no recurrence even up to 20 months of follow-up after endoscopic excision.

**Conclusion:**

Extracranial primary atypical meningioma should be kept in mind as one of the differential diagnoses of nasal mass. Histopathological diagnosis along with immunohistochemistry should be used for definitive diagnosis.

## 1. Introduction

Meningioma is a central nervous system tumor that typically arises in proximity to meninges. Rarely in less than 2% of the cases, meningiomas appear extracranially, especially in head and neck regions, sinonasal tract, ear, temporal bone, and scalp [1]. Histopathological and immunohistochemistry examination is usually diagnostic. WHO (World Health Organization) has classified meningioma into 3 grades: benign, atypical, and anaplastic [2]. Some studies have shown 30% of meningiomas to be atypical [3].

Here, we are presenting a case of primary atypical nasal meningioma.

## 2. Case Report

A 63-year-old female presented to our outpatient department with complaints of right-sided nasal obstruction and intermittent epistaxis for 6 months. Her nasal obstruction was insidious in onset, gradually progressive, and for the last 3 months, there was complete obstruction. Epistaxis was intermittent, 1 episode in a month, spontaneous, and 250–500 ml in amount in each episode. There was no history of any systemic diseases.

**Figure 1:**
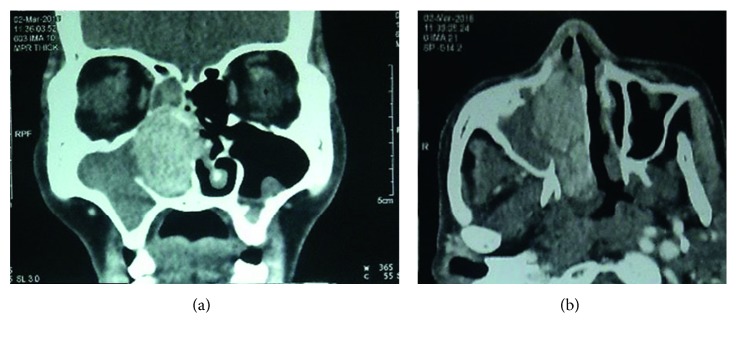
CECT nose and PNS (coronal and axial cut) showing heterogeneously enhancing mass in the right nasal cavity, likely centered in the middle turbinate with extension into the right ethmoid sinus superiorly, floor of nasal cavity inferiorly, choana and sphenoid sinus posteriorly with the erosion of the adjacent bone, and destruction of the nasal septum with extension of the mass to the contralateral side.

Examination of the nasal cavity revealed pinkish fleshy mass occupying the entire right nasal cavity that bled easily on touch. CECT (contrast-enhanced computed tomography) of the nose and paranasal sinuses (Figure 1) showed heterogeneously enhancing mass in the right nasal cavity, likely centered in middle turbinate with extension into the right ethmoid sinus superiorly and choana and sphenoid sinus posteriorly with erosion of the adjacent bone. There was destruction of the nasal septum along with extension of the mass to the contralateral nasal cavity, features suggestive of inverted papilloma.

**Figure 2:**
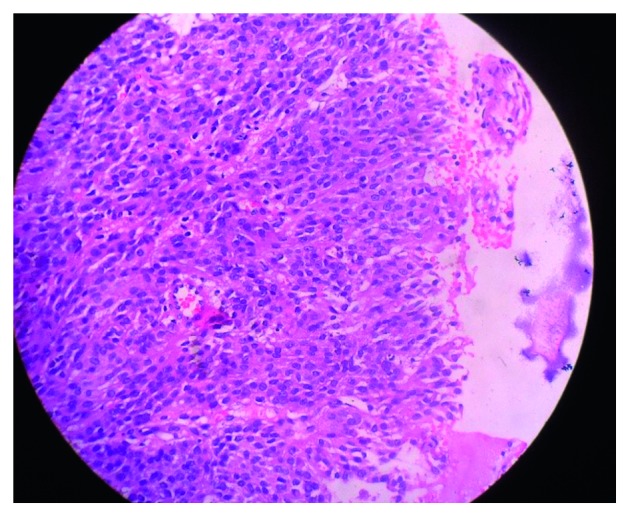
Histopathological examination suggestive of a WHO grade II atypical transitional meningioma (H&E stain).

To confirm the diagnosis, biopsy of the mass was taken. Histopathological examination (Figure 2) revealed unencapsulated tumor composed of tumor cells arranged in lobules, nests, and sheets with focal whorling pattern in subepithelium. The tumor cells were epithelioid to spindle-shaped with monomorphic round nuclei, moderate amount of eosinophilic cytoplasm, and indistinct cell borders. The chromatin was fine with occasional intranuclear inclusions and many prominent nucleoli. Some areas also showed spindling with oval nucleus arranged in short fascicles. Mitotic figures were present: 4 per 10 high-power field. Neither necrosis nor pigmentation was seen. Features were suggestive of a WHO grade II atypical transitional meningioma. Due to prominent nuclei and S100 positivity, HMB-45 was done which was negative, thus ruling out malignant melanoma.

**Figure 3:**
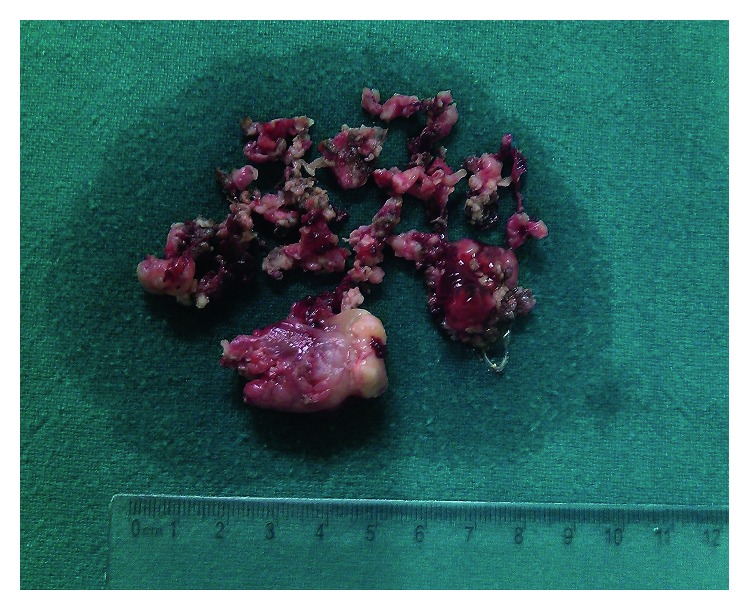
Meningioma after endoscopic excision.

Initially, preoperative internal maxillary artery embolization was planned, but catheterization could not be performed due to tortuosity of the right common iliac artery. She underwent endoscopic excision of the mass. Preoperatively, there was a pinkish, vascular, and friable mass occupying the right nasal cavity, extending anteriorly up to the anterior end of the right middle turbinate and posteriorly occupying the choana along with extension towards the left side (Figure 3). Superiorly, the mass was attached to the roof of ethmoid and inferiorly to the floor of the right nasal cavity. No destruction of septum was seen as suggested by CECT. There was no recurrence even after 20 months of follow-up.

## 3. Discussions

Meningiomas are nonglial tumors of the central nervous system, representing 24–30% of all intracranial neoplasms. They arise from the arachnoid cap cells (meningocytes) that are derived from the neural crest. These tumors have a predilection for females and have bimodal age distribution with first peak at the second decade of life and second peak during fifth through seventh decades [1, 4]. They have been reported to occur extracranially in only 1-2% of cases, and 20% of extracranial meningiomas are secondary extensions of intracranial tumors [5]. Primary extracranial meningiomas without direct communication with the intracranial region are rare. Histologically, primary extracranial meningiomas are identical to intracranial counterparts.

There are different mechanisms contributing to the development of the extracranial meningiomas. During embryogenesis, when arachnoidal cells that are present in the sheaths of nerves or vessels emerge through the skull foramina, the then displaced Pacchionian bodies are detached, pinched off, or entrapped in an extracranial location. It can also occur following a traumatic event or cerebral hypertension that displaces the arachnoid islets, originated from undifferentiated or multipotent mesenchymal cells, such as fibroblasts, Schwann cells, or a combination of these, perhaps explaining the diverse pathologic spectrum found in meningiomas [1, 6, 7].

Bassiouni et al. have classified primary extradural meningiomas into four types: Type I (epidural), tumor located between the dura mater and the inner calvarial table; Type II (calvarial or diploic), located between the outer and the inner calvarial table; Type III (extracranial), located outside the outer calvarial table; and Type IV (mixed), tumor extending from the dura to extracalvarial space [8].

They have been reported to occur in the sinonasal tract, cranial bones, middle ear, scalp, and soft tissues of the face and neck, and parotid gland. An analysis of 146 cases of primary extracranial meningiomas showed that the majority of them originated from the skin and scalp (*n* = 59) followed by middle ear (*n* = 26) and sinonasal tract (*n* = 25). Other rare locations in the head and neck are the temporal bone, mandible, nasopharynx, parotid gland, orbit, and neck [9].

Symptoms depend on the anatomic site of involvement. Meningiomas involving the sinonasal tract may mimic sinusitis with patients presenting with the nasal obstruction, anosmia, facial pain, nasal discharge, and epistaxis [5, 10]. Some authors describe that an average duration of extracranial sinonasal meningioma is 31.1 months. Nasal endoscopy usually shows a firm reddish pink to grey mass in the nasal cavity which could be globular or lobulated but well circumscribed with displacement and without infiltration into the surrounding tissues [1]. Radiological findings are usually nonspecific and include clouding or opacification of the sinuses, bony sclerosis, and focal destruction of the surrounding sinusoidal or nasal cavity bony tissues [11].

In the current WHO edition (2007), grade I meningiomas (benign) are recognized by their histologic subtype and lack of anaplastic features. Grade II meningiomas (atypical) are defined by one or more of the following four criteria: (1) chordoid or clear cell histologic subtype, (2) 4 to 19 mitoses per ten high-power field (HPFs), (3) brain infiltration, and (4) 3 or more of the following five histologic features: small cell change, increased cellularity, prominent nucleoli, sheet-like growth, or necrosis. Grade III meningiomas (anaplastic/malignant) are defined by rhabdoid or papillary subtypes, a histological picture of frank malignancy resembling that of carcinomas, melanomas, or high-grade sarcomas, or 20 or more mitoses per 10 HPFs [2]. A report of an atypical primary meningioma of nasal septum undergoing malignant transformation with distant metastasis into scalp and anterior chest wall can be found in literature [12].

In a study done in 163 cases, the most common histopathological subtype in primary extracranial meningiomas of head was meningothelial meningioma (53.4%) followed by transitional type (12.3%), psammomatous type (11.7%), and fibrous type (6.7%). Meanwhile microcystic, metaplastic, secretory, and regressive types were rare consisiting of 2.4% [4].

The differential diagnosis of sinonasal tract meningiomas includes mucocele, olfactory neuroblastoma, carcinoma, hemangioma, sarcoma, and angiofibroma [1, 10]. Histopathology and immunohistochemistry are confirmatory. Meningiomas are strongly immunoreactive to vimentin, EMA (epithelial membrane antigen), and pancytokeratin. Besides these, some meningiomas also show positive reactions to CK7, S100 protein, CAM 5.2, synaptophysin, CK20, GFAP, Ki-67 index (>1%), CD34, SMA, PCNA, progesterone receptor, and estrogen receptor [1, 9].

Studies have shown that the growth of the tumor is slow; hence, surgical extirpation without the necessity of adjuvant therapy is the treatment of choice, but complete excision is not always possible due to complex anatomy of nasal cavity and paranasal sinuses [1, 9]. Complete extirpation of these masses may be accomplished by endoscopic, open, or a combination of both approaches. Radiation therapy has been suggested to improve survival in meningiomas of central nervous system, but its role in extracranial meningiomas has not been studied yet. In our case, endoscopic surgery was done, and microdebrider along with monopolar cautery was used to excise the tumor in piecemeal due to vascular nature of the mass. In cases with high-risk tumor locations, stereotactic surgery has shown improved results. Studies have found that tumors ranging from 1 to 8 cm with an average of 3.5 cm usually infiltrate bone of the sinus or nasal cavity and an intact surface epithelium without ulceration or penetration [1].

In our case, there was no recurrence on subsequent follow-up of 20 months. Usually, recurrence occurs at the same anatomic site as the primary lesion, and depending on the time interval, it may be distinguished from the residual disease. A study analyzing the relationships between tumor sites and recurrence and death in 170 cases has found statistically significant difference among recurrence and type of removal. They found 9.7%, 14.3%, and 66.7% recurrence rates in total, subtotal, and partial removal cases, respectively. Further, they found death rates of 5.4% and 28.6% in total and subtotal removal cases, respectively. Recurrence was found to be 8% in benign meningioma, 11.1% in atypical, and 57.1% in malignant type [4]. Additional surgery, if clinically feasible, is advisable as radiation therapy does not always result in a clinical response.

A 5-year disease-free survival rate was found to be 66.9% to 82.1%, and 10-year disease-free survival rate was found to be 54.6% to 78.6% [1, 9]. Death rate of malignant (57.1%) is highest compared to atypical (11.1%) and benign (2.3%) [4].

## 4. Conclusions

Primary sinonasal atypical meningioma is a rare condition. It should be considered as one of the differential diagnoses of a nasal mass. Histopathological diagnosis along with immunohistochemistry should be used for definitive diagnosis. Excision can be done by endoscopic endonasal approach.
